# Efficient Wear Simulation Methodology for Predicting Nonlinear Wear Behavior of Tools in Sheet Metal Forming

**DOI:** 10.3390/ma15134509

**Published:** 2022-06-27

**Authors:** Junho Bang, Minki Kim, Gihyun Bae, Hong-Gee Kim, Myoung-Gyu Lee, Junghan Song

**Affiliations:** 1 Molding & Metal Forming R&D Department, Korea Institute of Industrial Technology, Incheon 21999, Korea; bjh@kitech.re.kr (J.B.); mkim@kitech.re.kr (M.K.); baegh@kitech.re.kr (G.B.); 2 Department of Materials Science and Engineering, Seoul National University & RIAM, Seoul 08826, Korea; 3 Materials Forming Research Group, POSCO Global R&D Center, Incheon 21985, Korea; hgkim5@posco.com

**Keywords:** wear simulation, sheet metal forming, advanced high-strength steel, wear test, tool wear

## Abstract

In conventional wear simulation, the geometry must be updated for succeeding iterations to predict the accumulated wear. However, repeating this procedure up to the desired iteration is rather time consuming. Thus, a wear simulation process capable of reasonable quantitative wear prediction in reduced computational time is needed. This study aimed to develop an efficient wear simulation method to predict quantitative wear reasonably in reduced computational time without updating the geometry for succeeding iterations. The wear resistance of a stamping tool was quantitatively evaluated for different punch shapes (R3.0 and R5.5) and coating conditions (physical vapor deposition of CrN and AlTiCrN coatings) by using a progressive die set. To capture the nonlinear wear behavior with respect to strokes, a nonlinear equation from a modified form of Archard’s wear model was proposed. By utilizing the scale factor representing the changes in wear properties with respect to wear depth as input, the simulation can predict the behavior of rapidly increasing wear depth with respect to strokes after failure initiation. Furthermore, the proposed simulation method is efficient in terms of computational time because it does not need to perform geometry updates.

## 1. Introduction

In recent years, the acceleration of the development of eco-friendly vehicles such as electric vehicles and hydrogen vehicles has led to a growing demand for lightweighting of the vehicle body to improve mileage. In addition, for passenger safety and the protection of batteries and fuel cells, many efforts have been made to secure the crashworthiness of the vehicle body. Automobile body weight can be reduced by using lightweight materials such as advanced high-strength steel (AHSS), aluminum alloys, magnesium alloys, glass-fiber composites, and carbon-fiber composites. Although AHSS results in the least weight reduction among these lightweight materials, it is relatively inexpensive to produce AHSS automotive body components [1,2,3]. Furthermore, a vehicle structure composed of AHSS showed outstanding crash safety performance in a simulation of three crash tests: frontal wall impact, side barrier impact, and roof strength tests [4]. To reduce the cost and meet safety regulations, automakers have increased the proportion of AHSS used in vehicle structures [5]. Moreover, to reduce the manufacturing cost of mass-produced vehicle components, the cold forming process is generally used. However, the cold forming of AHSS sheets is relatively vulnerable to tool wear. The higher strength characteristics of AHSS sheets in comparison with conventional steel sheets may generate premature tool wear, which leads to many problems such as reduced productivity, reduced product quality, and increased maintenance cost. Therefore, in the mass production of automobile components using AHSS sheets, the investigation of tool wear resistance is essential for efficient process management.

Owing to the problems with tool wear caused by the increase in the strength of steel sheets applied to the automobile body, several studies to delay or avoid the wear responses in sheet metal forming have been investigated. The sensitivity of various tooling and process parameters affecting the tool wear resistance have been studied. Pereira et al. [6] investigated the effect of machining error of the die profile shape on tool wear in sheet metal forming. A small and localized change in the die radius profile shape has a significant effect on tool-life reduction. Podgornik and Jerina [7] confirmed the effect of surface roughness on galling resistance for coarse and fine ground forming tools with a monolayer physical vapor deposition (PVD) TiN hard coating, and multilayer plasma-assisted chemical vapor deposition (PACVD) W-doped DLC coating under dry sliding conditions. Podgornik et al. [8] investigated the influence of the surface preparation condition of forming tool steel with PVD coatings (TiN, TiB_2_, TaC, and WC/C) on galling properties. Gonzalez-Pociño et al. [9] confirmed the heat treatment parameters (quenching, tempering, and nitriding process) that significantly affect adhesive wear resistance through an experiment in which these parameters were deliberately changed. Ogunbiyi et al. [10] suggested that the wear performance of Inconel 738 low-carbon composites could be enhanced with the addition of graphene nanoplatelets. Büyükkayacı et al. [11] investigated the influence of mechanical alloying time on wear resistance for Fe–Cu–C alloys. Sıralı et al. [12] determined the effect of grain size reduction of titanium–zirconium–molybdenum alloys obtained with the addition of Ti on wear performance. Woodward et al. [13] investigated the sliding wear response of G320 grey cast iron in different microstructure conditions resulting from quench and temper heat treatment. Hou et al. [14] evaluated the galling behaviors in sheet metal forming under various tool conditions with respect to hardness, surface roughness, and coating. Ghiotti and Bruschi [15] and Cora et al. [16] investigated the effect of various coating techniques on wear resistance by using a wear test system imitating stamping process conditions. van der Heide et al. [17] evaluated the effects of various lubricated conditions on galling by using a slider-on-sheet tribometer.

Although the wear resistance of a tool can be improved by controlling various tooling and process parameters affecting the tool wear resistance as presented in the papers described above, they are not sufficient to predict the evolutionary behavior of tool wear and the initiation of tool failure. An experimental method to estimate the wear resistance of a tool can enable the prediction of the tool lifetime and facilitate effective management of stamping tools in mass production. Therefore, extensive experimental methods have been proposed for estimating the wear of stamping tools in the sheet metal forming process. Bang et al. [18] performed a pin-on-disk test based on the Taguchi method to construct a wear prediction model in sheet metal forming. However, it has the disadvantage that the pin repeatedly contacts the already worn sheet material surface. To compensate for this shortcoming, slider-on-sheet–type wear test methods, in which the tool comes into contact with the virgin sheet material, have been used [19,20,21,22,23]. However, the wear test methods described above do not reflect the deformation modes encountered in automotive sheet metal forming. Thus, representative wear tests considering the actual conditions prevalent in automotive sheet metal forming were performed, such as U-bending [6,24,25,26,27,28], bending under tension [29,30,31,32,33], and deep drawing [34]. However, they are cumbersome and uneconomical to perform on stamping tools with a large number of strokes. A wear test should be fast, convenient, efficient, and economical to investigate the wear characteristics of a stamping tool and reflect the real stamping conditions prevalent in automotive sheet metal forming. Bang et al. [35] proposed a systematic wear test methodology using a progressive die set in sheet metal forming. This apparatus was designed to conduct wear evaluation of four types of punches simultaneously with a single press stroke. Although various experimental methods have been presented for evaluating the tool wear in sheet metal forming, the wear evaluation of the press forming tool still has limitations in that it entails significant cost, time, and human effort. 

To reduce the time required for tool wear prediction in sheet metal forming, several numerical simulation studies have been conducted on tool wear prediction. Hoffmann et al. [36] proposed a wear simulation scheme using Archard’s wear equation to calculate the elemental wear, which is linearly proportional to the number of strokes. However, in sheet metal forming, the tool wear is nonlinear with respect to the number of strokes. Therefore, many efforts have been made to estimate the wear behavior of stamping tools reasonably and accurately. Ersoy-Nürnberg et al. [37] proposed a modified Archard’s model with a variable wear coefficient with respect to the loading duration. The wear coefficient is a function of the accumulated wear work and is determined through deep-drawing experiments. Wang et al. [38] approximated the changes in wear coefficient during testing by using pin-on-disk test results to reflect the variation of wear characteristics reasonably. The studies described above used a simulation scheme of updating the geometry based on changes to the geometry calculated from interactive iterations of forming and wear simulation. A wear simulation calculates elemental wear using contact-related information obtained from a forming simulation. The updated geometry from the wear simulation is used in the next forming simulation. These procedures are repeated until the desired iteration. Therefore, the conventional wear simulation scheme considering the geometry update requires substantial computational time to predict the wear of forming tools. In addition, because the studies described above evaluated only simple models, wear simulation for complex and large parts such as automobile components requires massive computational time. Therefore, it is necessary to establish a reasonable wear simulation process that takes less computational time and considers the nonlinear tool wear behavior in sheet metal forming.

The present study developed a wear simulation procedure capable of predicting the nonlinear wear behavior of stamping tools in reduced computational time. A nonlinear equation from a modified form of Archard’s wear model was constructed based on the wear test results of a stamping process for different punch shapes (R3.0 and R5.5) and coating conditions (PVD CrN and AlTiCrN coatings). The scale factor, which represents the changes in wear properties with respect to wear depth, was utilized in the wear simulation to avoid the update of geometry from the previous iteration of wear simulation. By formulating a wear coefficient of Archard’s wear model as a function of strokes and implementing the wear coefficient into the scale factor of wear simulation, the nonlinear wear behavior of the stamping tools could be estimated. Therefore, the suggested wear simulation method can save computational time in the prediction of the nonlinear wear behavior of stamping tools.

## 2. Wear Test

### 2.1. Materials

The steel sheet used in this work was an uncoated transformation-induced plasticity (TRIP) steel sheet with a tensile strength of 1180 MPa (TRIP1180) and a thickness of 1.2 mm, which was produced by POSCO Pohang steelworks. The uniaxial tensile properties of a 1.2-mm-thick TRIP1180 steel sheet were obtained from previous work [35], which is summarized in Table 1.

**Figure 1:**
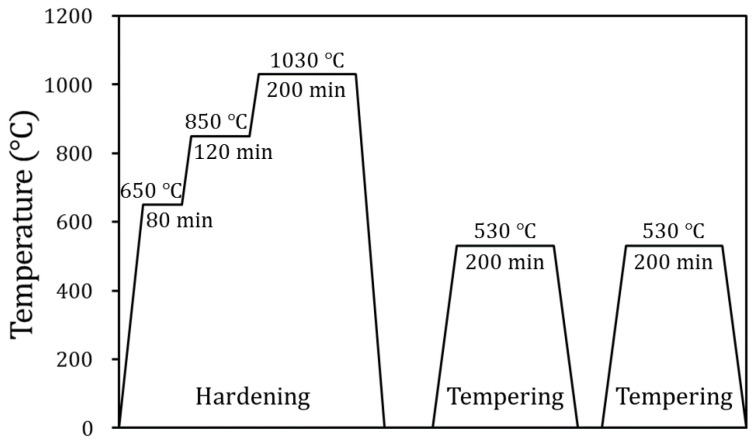
Heat treatment conditions for STD-11 tool steel.

The punch for forming the steel sheet during the wear test was composed of STD-11 tool steel (KS D 3753) manufactured by POSCO Pohang Steelworks. STD-11 tool steel is generally used for materials of stamping tools in the automotive industry and is comparable to SKD-11 (JIS G 4404), D2 (ASTM A 681-08), and 1.2379/X153CrMo12 (DIN EN ISO 4957). The mechanical properties of D2 tool steel are summarized in Table 2 [39]. In order to harden the STD-11 tool steel by introducing a martensite microstructure, heat treatment was performed as shown in Figure 1. The STD-11 tool steel was hardened in avvacuum at 650, 850, and 1030 °C for 80, 120, and 200 min, respectively, and then air-cooled. A two-step tempering was performed at 530 °C for 200 min to reduce brittleness caused by quenching. The Vickers hardness at a 0.1 kgf normal load of the STD-11 tool steel was 788.5 ± 12.2 HV_0.1_, which was referred to in previous literature [35]. The chemical compositions of TRIP1180 steel and STD-11 tool steel are listed in Table 3.

To quantitatively evaluate the wear lifetime with respect to coating conditions, CrN and AlTiCrN coatings were used for the wear tests and deposited on the STD-11 tool steels using PVD. CrN coatings are commonly used for the tools in press forming. AlTiCrN coatings are known to provide excellent wear resistance in the forming of ultra-high-strength steel (UHSSs) sheets. The Vickers hardness at a 0.08 kgf normal load of the CrN and AlTiCrN coatings was 2105.9 ± 15.5 and 3818.3 ± 36.5 HV_0.08_, respectively, and the roughness of the punch coated with CrN and AlTiCrN was 0.22 ± 0.04 and 0.23 ± 0.03 μm, respectively, which were also referred to previous literature [35].

### 2.2. Experimental Setup

**Figure 2:**
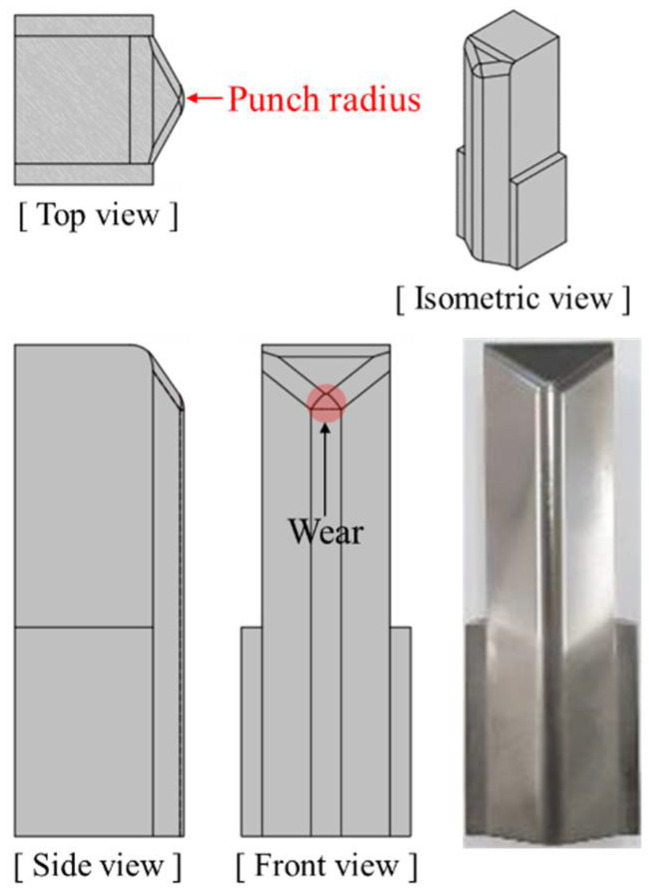
Punch geometry used for the wear test, which is designed to localize wear to the punch radius, as proposed by Bang et al. [35].

In automotive sheet metal forming, tool wear generally occurs in the sharp-curvature area of tools subjected to localized contact pressure. The tool used for the wear test imitated the sharp-curvature geometry prevalent in automotive sheet metal forming. A smaller curvature radius of the forming tool causes a higher contact pressure to be localized on the tool surface. Thus, to evaluate the sensitivity of wear rate according to the tool shapes, a punch with a radius of 3.0 mm and 5.5 mm was introduced for the wear test and the wear of both punches was designed to be localized in the curvature regions. The punch geometry is presented in Figure 2. Dimensions of both punch shapes are previously reported in detail in the literature [35]. Die-to-punch gap was defined as 1.32 mm to avoid ironing, which is 10% clearance. Furthermore, they were designed as insert-type punches to efficiently inspect and interchange worn-out punches. The punch stroke for one stroke was 35.0 mm. The geometrical parameters for the stamping process were the same as in a previous report.

**Figure 3:**
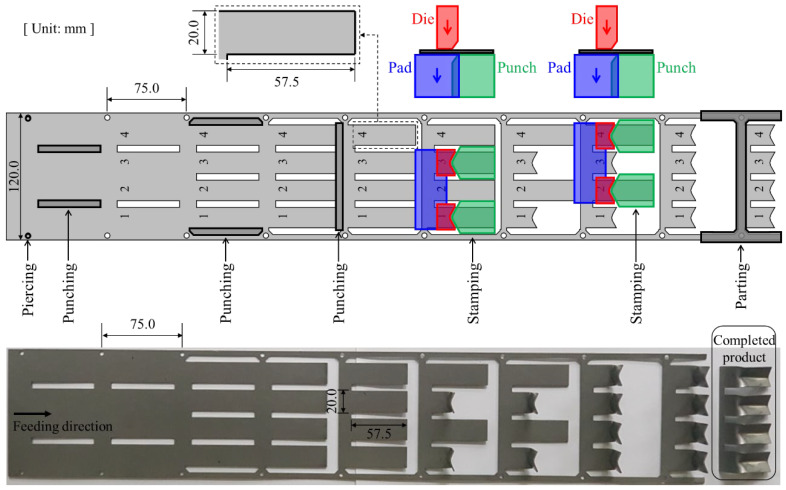
Layout of metalworking operations for the progressive die tool [35].

In general, when forming UHSS, several thousand to tens of thousands of strokes are required to examine the wear characteristics of coated tools. Thus, in the present study, progressive die tools are suitable for continuous wear tests. A press system, uncoiler, and automatic feeding system were used for the fast, convenient, and systematic wear test. Because a rolled-steel coil was directly used without any production of the specimen, it was more economical and efficient. The layout for the various metalworking operations of the employed progressive die tool is shown in Figure 3. The progressive die tool was designed to test the four types of punches simultaneously, thereby reducing the time and cost required for the wear tests. Metalworking operations involve techniques such as piercing, punching, stamping, and parting. The piercing process creates holes in the sheet metal by using a shearing tool. These holes allow the sheet metal to move into a progressive die set in sections of 75.0 mm during metalworking operations. The punching process entails the creation of a blank shape by using a shearing tool. The continuous stamping process causes wear on the punch surface due to the contact between the punch and blank. The blank created through the punching process was 20.0 mm and 57.5 mm in width and length, respectively. The press rate was 15 strokes per minute. In the parting process, the formed products are cut from sheet metal by using a shearing tool. A detailed description for each metalworking operation and testing setup has been reported in a previous paper [35] that proposed the test method of tool wear in sheet metal stamping.

Gradual damage to the punch surface during the press forming process leads to a change in the surface profile of the punch. Thus, the wear depth of the punch surface was quantitatively evaluated with respect to strokes. The surface profile of the curvature regions where the wear is localized was measured before and after the wear test. Punch surface profilometry was conducted using a contact-type three-dimensional (3D) coordinate measuring machine. The measurement methods of wear depth have been described elsewhere in detail [35].

The wear resistance of stamping tools was quantitatively evaluated for two punch shapes with radii of 3.0 mm and 5.5 mm. To compare the wear resistance according to the coating conditions, the wear lifetime of CrN- and AlTiCrN-coated punches was quantitatively evaluated for the stamping process. CrN and AlTiCrN coatings were deposited on steel tool die (STD)-11 tool steel using physical vapor deposition (PVD). The punch conditions of the wear test performed in this study are summarized in Table 4.

### 2.3. Wear Test Results

**Figure 4:**
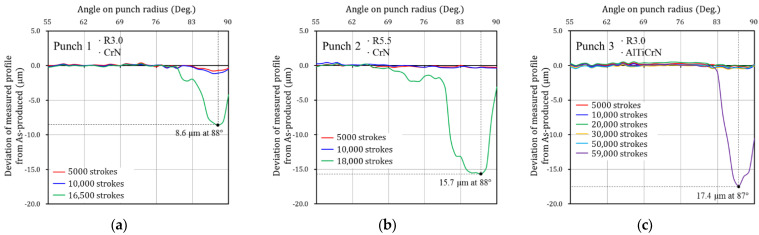
Evolutionary behavior of wear depth on the punch radius with respect to the number of strokes: (**a**) Punch 1 (PVD CrN-coated punch with a radius of 3.0 mm), (**b**) Punch 2 (PVD CrN-coated punch with a radius of 5.5 mm), and (**c**) Punch 3 (PVD AlTiCrN-coated punch with a radius of 3.0 mm).

In sheet metal forming, failure can be determined from visible scratches and rough wear tracks on the surface of the formed products [35,40,41]. To identify the evolutionary behavior of wear of the stamping tool, the wear depth was measured for each punch at the same number of strokes and wear tests were performed until failure occurred on the product surface formed with each punch. Wear tests for Punch 1, Punch 2, and Punch 3 were performed up to 16,500, 18,000, and 59,000 strokes, respectively. For all three punches, the deviation of the measured profile from the As-produced condition is very small and is within the measurement error before failure. However, after failure, wear occurred at approximately 80°–90° on the punch radius for all punches, and the wear depth increases rapidly at the wear area, as shown in Figure 4.

In the case of Punch 1 (CrN-coated punch with a radius of 3.0 mm), failure occurred after 16,500 strokes. The wear regions on the punch radius ranged from 81° to 90° and the maximum wear depth was 8.6 μm at 88° on the punch radius, as shown in Figure 4a. For Punch 2 (CrN-coated punch with a radius of 5.5 mm), failure occurred after 18,000 strokes. The wear was found from 80° to 90° on the punch radius and the maximum wear depth was 15.7 μm at 87° on the punch radius, as shown in Figure 4b. Punch 1 with a radius of 3.0 mm failed 1500 strokes earlier than Punch 2 with a radius of 5.5 mm because the former had a higher contact pressure than the latter. In the case of Punch 3 (AlTiCrN-coated punch with a radius of 3.0 mm), wear regions on the punch radius ranged from 84° to 90° of the punch radius, and a maximum wear depth of 17.4 μm occurred at 87° on the punch radius after 59,000 strokes, as shown in Figure 4c. The wear resistance of punches coated with CrN and AlTiCrN with the same shape (R3.0) was quantitatively evaluated. Punch 3 (AlTiCrN-coated punch) exhibited wear response at 59,000 strokes and, thus, demonstrated significantly improved wear resistance over Punch 1 (CrN-coated punch) which failed at 16,500 strokes because the AlTiCrN coating (3818 ± 36.5 HV_0.08_) has a higher hardness than the CrN coating (2105 ± 15.5 HV_0.08_). The wear characteristics and mechanism of the punches were described in detail in previous literature [35].

## 3. Forming Simulation

An FE simulation was conducted to predict the contact conditions on the punch surface during the stamping process, which was performed using LS-Dyna R11.0 explicit code [42]. To reduce the computational time, the simulation was simplified to a half model using symmetric boundary conditions. Ersoy-Nürnberg et al. [37] reported that the elastic deformation of a tool is not significantly correlated to the contact pressure distribution on the tool surface. On the other hand, Pereira et al. [24] confirmed that the analytical rigid tool model (E = ∞) and the elastic tool model (E = 205 GPa) were compared to confirm the effect of the elasticity of the tool material on the contact pressure. The maximum contact pressure of the analytical rigid tool solution was predicted to be 30% higher than that of the elastic tool solution. According to the literature [43,44], the elastic modulus of the CrN and AlTiCrN coatings are 400.0 and 469.5 GPa, respectively, so the difference in maximum contact pressure between the tool model to which the elastic modulus of the coating is applied and the analytical rigid tool model is expected to be less than 30%. Moreover, for wear simulation of complex and large parts such as automobile components, the tools modelled with deformable solid elements require significant computational time, so rigid-body modelling of the tools is usually assumed in order to simplify the simulation model. In this sense, the tools (punch, pad, and die) were modelled with rigid bodies. They also demonstrated that when a blank was modelled with shell and solid elements, the wear simulation results for both element types were in excellent agreement with the experimental results. Thus, the blank was discretized by deformable shell elements, which also helps to reduce the computational time. 

**Figure 5:**
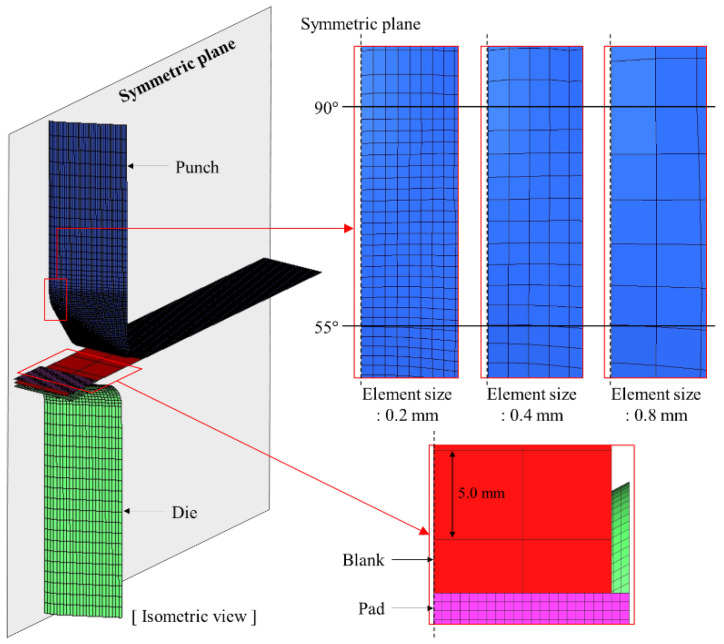
FE modeling to evaluate mesh size sensitivity at the stamping process contact interface.

The contact characteristics on the tool surface are sensitive to the mesh quality. Thus, the sensitivity of the contact interface mesh size to the contact pressure was confirmed for the punch geometry with a radius of 3.0 mm. The mesh size at the punch contact interface was evaluated for 0.2 mm, 0.4 mm and 0.8 mm. To reduce computational time, the mesh refinement method was applied to the simulation model. The initial blank mesh size was 5.0 mm. By setting the mesh refinement method to 6, 5, and 4 levels, the final blank mesh size was 0.156, 0.313, and 0.625 mm for each punch mesh size case, respectively. Pad and die contact pressure prediction does not require accuracy, thus it was modelled with a relatively coarse size of 0.5 mm. FE modeling for the simulation is shown in Figure 5 with the FE model details summarized in Table 5. 

The number of integration points in the thickness direction of the blank was set to 5. Pereira et al. [24] confirmed that a Coulomb friction coefficient of 0.15 was appropriate to predict an accurate contact pressure distribution by comparing experiments and numerical simulations of the channel forming process. In this study, a Coulomb friction coefficient of 0.15 was assumed at the contact interface between the punch and blank because it is difficult to calculate the appropriate friction coefficient by measuring the punch reaction force of the stamping process among the various metalworking operations of the employed progressive die tool. Amounts of contact pressure generated on the contact interface are affected by the strength of the steel sheet. As shown in Table 1, the 1.2-mm-thick TRIP1180 steel sheet shows anisotropic characteristics. Therefore, Hill’s 1948 yield criteria [45] were employed to consider the anisotropic plastic deformation behavior and to predict the accurate contact pressure. The anisotropic parameters of the Hill’s 1948 yield criteria used in the simulation are summarized in Table 6. TRIP1180 with a thickness of 1.2 mm was used as the blank material, as summarized in Table 1. The padding force was 12.0 kN, which is sufficient to hold the sheet metal during the stamping process. The punch stroke was 35.0 mm.

**Figure 6:**
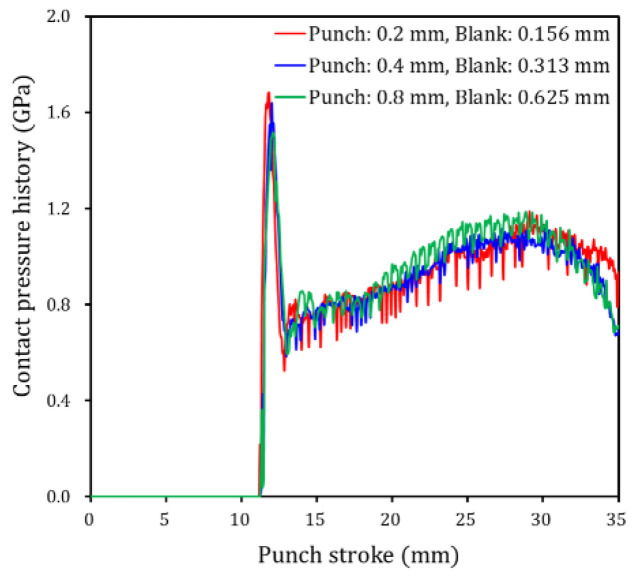
Contact pressure history comparison at a node on the punch radius where the highest contact pressure occurs to evaluate contact characteristics according to element sizes.

Figure 6 compares the contact pressure history at a node on the punch radius where the highest contact pressure occurs to evaluate contact characteristics according to mesh sizes and predicted contact pressures for all mesh size cases are of similar magnitude. The simulation was performed on a computer with an Intel Core i7-6700 CPU and 8 GB RAM. Computational time was approximately 8 h, 2 h, and 20 min, respectively, as summarized in Table 5. Thus, a mesh size of 0.8 mm at the punch contact interface can effectively reduce computational time. However, as shown in Figure 5, if the mesh size at the contact interface is coarse, the number of elements between 55 and 90° on the punch radius is insufficient such that it is difficult to accurately analyze contact characteristics over the punch radius. Therefore, in the simulation cases for this study, a 0.2 mm mesh size was applied at the punch contact interface, and 0.156 mm was used for the final blank mesh size by applying six levels of the mesh refinement method, which is sufficiently refined for accurate prediction of the contact conditions on the punch surface.

**Figure 7:**
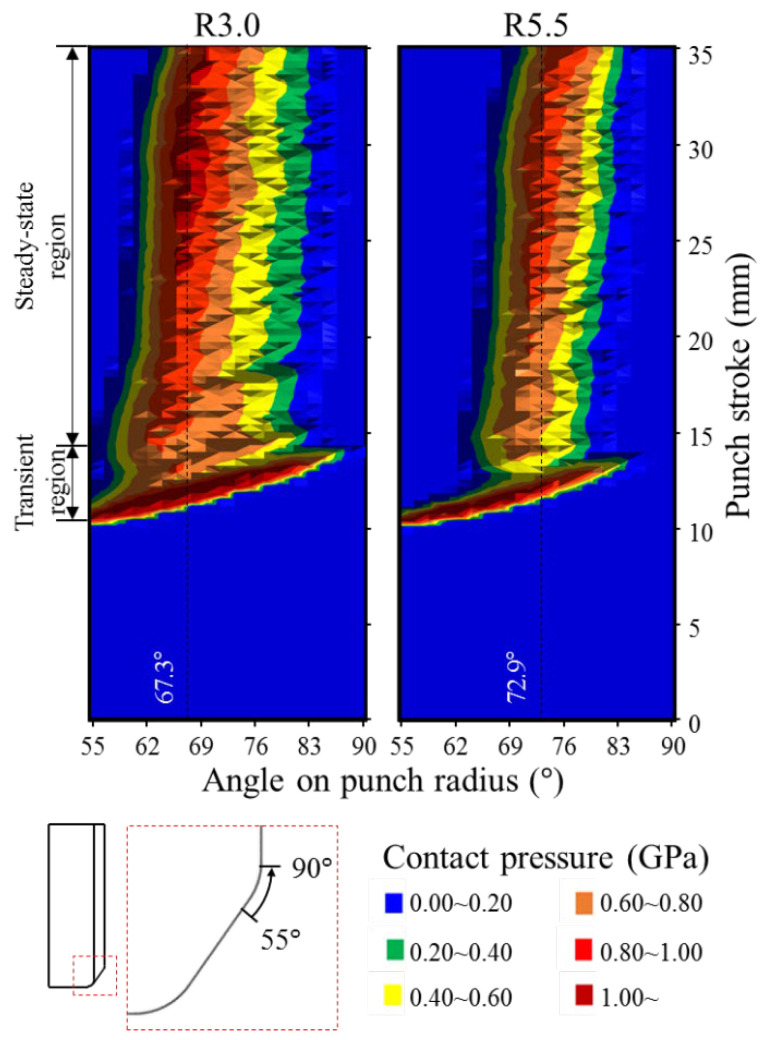
Time-dependent evolution of contact pressure on the punch surface during the stamping process.

**Figure 8:**
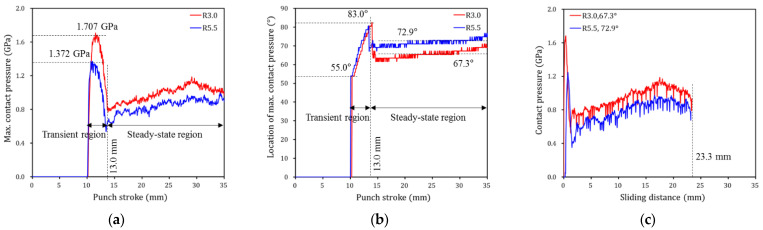
Contact conditions on the punch radius for the R3.0 and R5.5 punches: (**a**) maximum contact pressure with respect to the progression of the punch stroke, (**b**) location of the maximum contact pressure with respect to the progression of the punch stroke, and (**c**) contact conditions at approximately 67.3° and 72.9° on the punch radius for the R3.0 and R5.5 punches, respectively.

In sheet metal forming, the contact pressure and sliding distance are the most significant factors influencing tool wear [24,46,47,48,49]. Thus, these two factors on the punch radius were investigated. To understand the time-dependent evolution of contact pressure along the punch radius during the stamping process, Figure 7 shows contour plots of the contact pressure over the punch radius during stamping. The ordinate represents the punch stroke level during stamping, while the abscissa represents the angle on the punch radius. As shown in Figure 7, the contact pressure levels over the radius of the R3.0 punch were higher than that over the radius of the R5.5 punch. Figure 8a plots the maximum contact pressure with respect to the punch stroke level for each punch shape. A highly localized contact pressure was distributed on both punch shapes between approximately 10.0 mm and 13.0 mm of the punch stroke. In this punch stroke range, the maximum contact pressures acting on the R3.0 and R5.5 punches were 1.707 GPa and 1.372 GPa, respectively. These maximum contact pressures moved along the punch radius transiently from 55.0° to approximately 83.0° for both the R3.0 and R5.5 punches, as shown in Figure 8b, which plots the angular location of the maximum contact pressure as the punch stroke progresses. A relatively high contact pressure was experienced over most of the angular range of the punch radius during the short punch strokes. Pereira [24] defined the initial region of the stamping process as a transient region where the angular location and magnitude of the contact pressure on the punch radius change significantly; this region is between approximately 10.0 and 13.0 mm of the punch stroke in the present study. In this transient region, a blank was formed and wrapped over the punch radius such that a transient change in the contact pressure response occured with the changes in the contact conditions of the punch radius. As shown in Figure 7, a steady and relatively lower contact pressure than that of the transient region was distributed over the punch radius for both the R3.0 and R5.5 punches between approximately 13.0 mm and 35.0 mm of the punch stroke. Pereira [24] referred to the region with such a steady contact pressure response as the steady-state region. Figure 8a illustrates the magnitude of the maximum contact pressure in this punch stroke range in more detail. A relatively steady contact pressure was observed for both punches. In addition, the contact pressure of the R3.0 punch was approximately 0.15 GPa higher than that of the R5.5 punch. In the steady-state region, the relatively high contact pressure for each punch stroke was concentrated at approximately 67.3° and 72.9° on the punch radius for the R3.0 and R5.5 punches, respectively, as shown in Figure 8b. Although the geometrical wrapping angle of the blank was 90° for both punches, the contact angle on the punch radius of the R5.5 punch was larger. The difference in contact angle on the punch radius can be inferred from the geometrical difference between the R3.0 and R5.5 punches. Bang et al. [18] revealed that the wear of a coated tool is caused by the interaction between the contact pressure and the sliding distance in the stamping process. Therefore, the transient region, where the highly localized contact pressure and short sliding distance occur, is not considered to have a critical effect on the wear response. Therefore, tool wear is expected to occur in the steady-state region. Figure 8c shows the contact conditions at approximately 67.3° and 72.9° on the punch radius for the R3.0 and R5.5 punches, respectively, where the highest contact pressure and longest sliding distance in the steady-state region occur. Because the curvature of the R3.0 punch was sharper than that of the R5.5 punch, the R3.0 punch has a higher contact pressure.

## 4. Tool Wear Prediction Model

Archard’s wear model [47] is the most widely used model to predict the tool wear in sheet metal forming, and it is also built into LS-Dyna, as expressed in Equation (1):

(1) $$w=k\frac{pl}{H},$$

In Equation (1), the wear depth w is directly proportional to the contact interface pressure p and the relative sliding distance  l on the contact interface, and it is inversely proportional to the hardness of the wearing material H. k is a dimensionless wear coefficient. Equation (1) needs to be discretized in the elemental form to be implemented in FE simulations. The wear depth change $\dot{w}$ at a certain time t can be calculated as follows:

(2) $$\dot{w}=k\frac{p_{t}v_{t}}{H},$$

where $p_{t}$ is the contact interface pressure at a certain time t and $v_{t}$ is the sliding velocity at a certain time t. By integrating Equation (2) over time duration of the stamping process, the wear depth  w on the contact interface can be calculated as follows:

(3) $$w=\frac{k}{H}\int \left(p_{t}v_{t}\right)dt.$$

**Figure 9:**
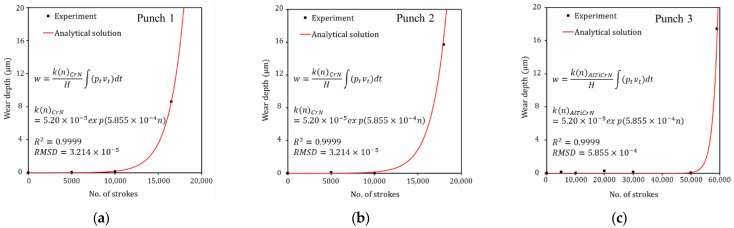
Definition of dimensionless wear coefficient k(n) of the analytical solution (Equation (4)) to accurately predict the evolutionary wear behavior of experimental wear data: (**a**) Punch 1 with a radius of 3.0 mm coated with PVD CrN, (**b**) Punch 2 with a radius of 5.5 mm coated with PVD CrN, and (**c**) Punch 3 with a radius of 3.0 mm coated with PVD AlTiCrN.

Because the wear depth was close to 0 before failure initiation, the contact pressure and the sliding distance on the contact interface were considered to be constant. Therefore, the wear depth predicted by Equation (3) is linear. As shown in the wear test results in Figure 4, the evolutionary wear behavior with respect to the strokes is not linear. Therefore, a nonlinear equation from a modified form of Archard’s wear model is proposed as follows:

(4) $$w=\frac{k\left(n\right)}{H}\int \left(p_{t}v_{t}\right)dt,$$

where k(n) is the dimensionless wear coefficient as a function of the number of strokes n. Here, k(n) is a variable that can express the wear behavior with respect to the number of strokes. The wear test results in Figure 4 show that wear depth is close to 0 before failure. When failure occurs, the wear depth increases rapidly. In order for the analytical solution (Equation (4)) to express the evolutionary wear behavior before and after failure and accurately simulate the experimental results (Figure 4), it is necessary to define k(n) for each punch. An exponential function was used for k(n) to approximate the rapidly increasing wear behavior with respect to strokes after failure initiation. If k(n) of Equation (4) is not defined, the wear behavior with respect to strokes is predicted linearly. As shown in Figure 9, it can be confirmed that the analytical solution (Equation (4)) accurately predicts the experimental wear data due to the definition of k(n). The fitted dimensionless wear coefficient k(n) for CrN- and AlTiCrN-coated punches are as follows:

(5) $$k{\left(n\right)}_{CrN}=Aexp\left(Bn\right)=5.20\times 10^{-5}exp\left(5.855\times 10^{-4}n\right),$$

(6) $$k{\left(n\right)}_{AlTiCrN}=Aexp\left(Bn\right)=3.05\times 10^{-15}exp\left(5.855\times 10^{-4}n\right).$$

The *R-squared* ($R^{2}$) is equal to 0.9999 for both exponential regressions. The *Root Mean Square Deviation* (*RMSD*) is $3.214\times 10^{-5}$ and $5.855\times 10^{-4}$ for each exponential regression, respectively. A of k(n) is related to the delay of the failure initiation, and B of k(n) means the rapidly increasing wear behavior after failure. In this study, the same value of $5.855\times 10^{-4}$ was assumed for B of k(n) due to the insufficient wear depth results to describe the wear behavior after failure initiation. The k(n) of the AlTiCrN-coated punch is smaller than that of the CrN-coated punch, which means that as the wear test progressed, the wear rate of the AlTiCrN-coated punch was slower. Thus, it is possible to simulate a failure initiation behavior occurring later in the AlTiCrN-coated punch.

The application of the constructed tool wear prediction model to wear simulation is described in detail in the next section.

## 5. Wear Simulation

**Figure 10:**
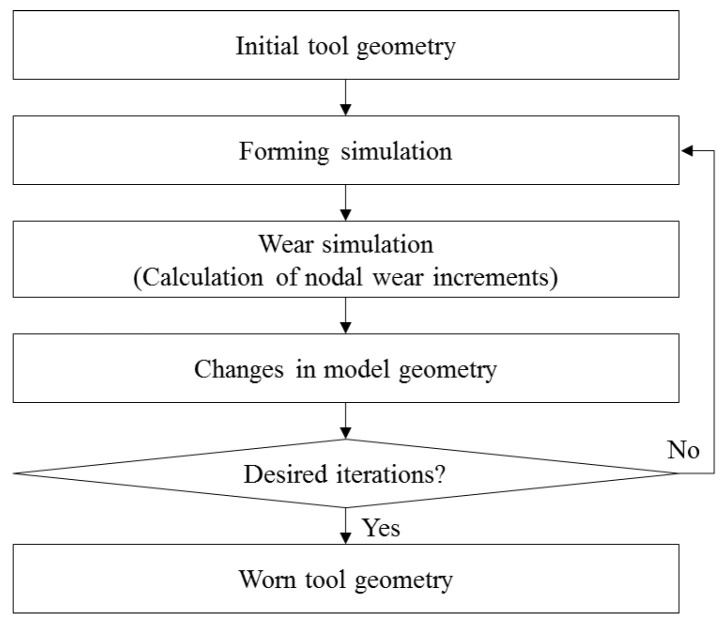
Process of wear simulation based on the modified Archard’s wear model.

Based on the constructed wear equation, a wear simulation was performed by following the process shown in Figure 10. First, the wear-related variables, hardness H, and dimensionless wear coefficient k, were entered in the “CONTACT_ADD_WEAR” card of the simulation keyword file. The hardness H is a value corresponding to each coating hardness. Although the dimensionless wear coefficient k is a function of the number of strokes n, as expressed in Equations (5) and (6), a value of 1 is set, as described in detail later. By performing numerical analysis with the constructed input file, the wear depth $w_{0}$ from Equation (4) is computed. Because k(n) is set to 1, the evolutionary wear behavior with respect to the number of strokes is not taken into account in the wear depth information. The computed wear depth information is contained in the dynain file. To perform the wear simulation and initialize the wear depth for the wear simulation process, the dynain file with wear depth information is included in the original input file. To predict the tool wear behavior, continuously track the wear status, and visualize the worn tool geometry with respect to the number of strokes, the “Wear analysis” application of LS-PrePost was used by reading in the input file including the dynain file. The scale factor F in the “Wear analysis” application was used to define the rapid increase in wear depth after failure initiation and predict the evolutionary wear behavior with respect to the strokes. The scale factor F is defined as a change in wear properties and characteristics with respect to the wear depth as wear progresses and can be explained by the difference in wear depth between *n* + 1 and *n* strokes, as expressed as follows:

(7) Scale factor (F)=k(n+1)−k(n).

**Figure 11:**
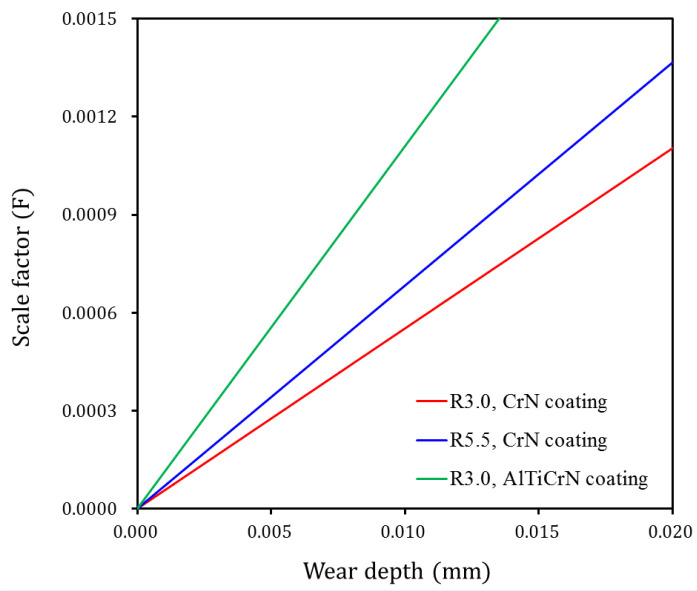
Scale factors for each punch condition.

Figure 11 plots the scale factors F for each stamping punch condition. The abscissa is the analytical solution for the wear depth calculated from Equation (4) for each stamping punch condition. The ordinate is the change in the dimensionless wear coefficient k(n) with respect to the change in the number of strokes n. In the case of the CrN-coated punches, the $k{\left(n\right)}_{CrN}$ (red line) of the R3.0 punch is smaller than that (blue line) of the R5.5 punch at the same wear depth value. That is, for punches with the same surface hardness, to calculate the same wear depth, k(n) is inversely proportional to the contact pressure and sliding distance, which can be calculated from Equation (4). Therefore, the scale factor of the R5.5 punch, which has a smaller contact pressure, is larger than that of the R3.0 punch. In the case of punches with the R3.0 punch shape, the $k{\left(n\right)}_{AlTiCrN}$ (green line) of the AlTiCrN-coated punch is larger than the $k{\left(n\right)}_{CrN}$ (red line) of the CrN-coated punch. For punches subjected to the same contact conditions, it can be confirmed from Equation (4) that k(n) is proportional to the surface hardness to calculate the same wear depth. This phenomenon is reflected in the simulation by inputting the wear depth versus scale factor F into the “Wear analysis” application in the piecewise linear format. The wear depth $w_{i}$ from i-th strokes can be calculated by multiplying the scale factor F by the wear depth $w_{0}$ from the forming simulation, and the amount and location of the punch wear can be visually confirmed in LS-PrePost. Therefore, this wear simulation method using the scale factor F does not need to update the geometry and calculate the accumulated wear from the previous iteration of the wear simulation.

**Figure 12:**
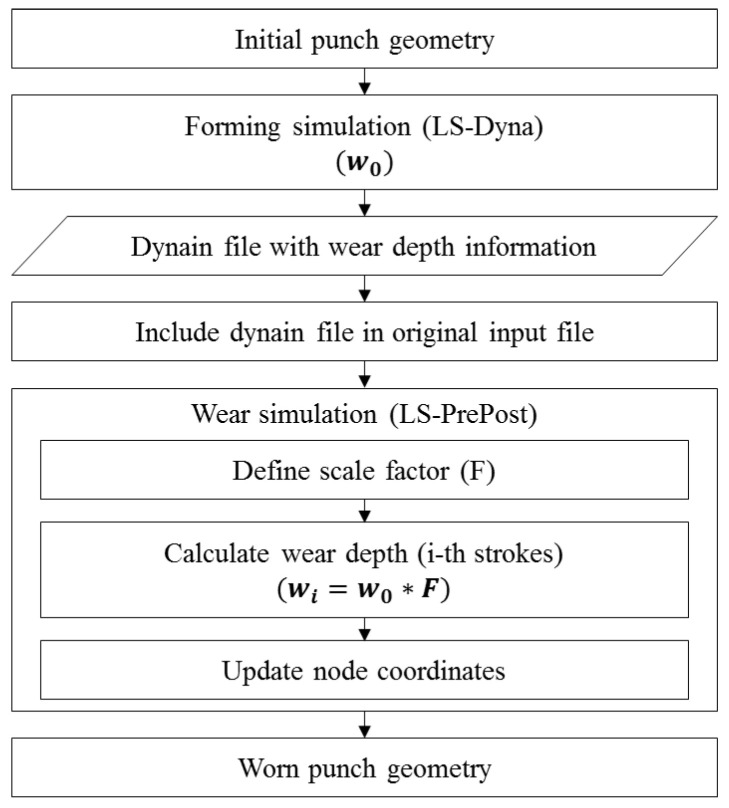
Conventional wear simulation method [37,38,50].

As shown in Figure 12, in the conventional wear simulation, the modified geometry should be updated from the geometry predicted in the previous iteration of the wear simulation to predict the accumulated wear amount up to the desired number of strokes. This process takes a large amount of time to repeat up to the desired iteration. In contrast, the proposed wear simulation procedure, using a scale factor  F representing the change in wear properties with respect to the wear depth, eliminates the need for repeating geometry updates from previous iterations of the wear simulation.

It was assumed that the contact pressure, sliding distance, and hardness on the contact interface of the punch were constant because the wear depth was close to 0 before failure initiation. This assumption was considered to be appropriate from the experimental results as shown in Figure 4. However, the wear depth just before failure initiation was not measured for all punches, so the constructed wear equation may not be accurate in predicting the wear depth just before failure initiation and it is necessary to verify the wear behavior just prior to failure for more accurate wear prediction.

## 6. Verification of Wear Simulation

**Figure 13:**
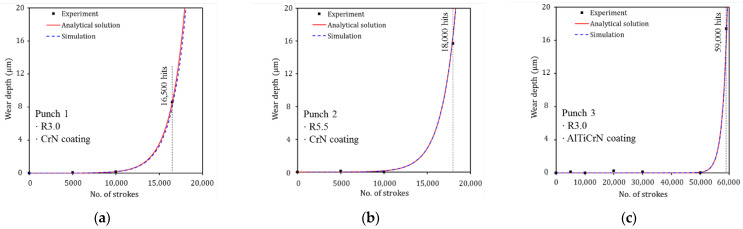
Comparison of the experimental results, analytical solution, and simulation results based on the revised Archard’s wear model with respect to the number of strokes: (**a**) Punch 1 with a radius of 3.0 mm coated with PVD CrN, (**b**) Punch 2 with a radius of 5.5 mm coated with PVD CrN, and (**c**) Punch 3 with a radius of 3.0 mm coated with PVD AlTiCrN.

**Figure 14:**
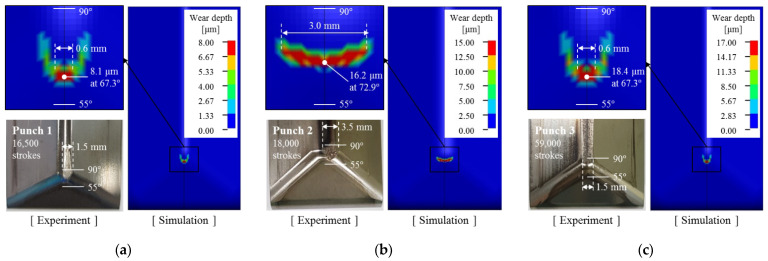
Predicted wear distributions and worn surface conditions on the punch surface after failure: (**a**) Punch 1 with a radius of 3.0 mm coated with PVD CrN, (**b**) Punch 2 with a radius of 5.5 mm coated with PVD CrN, and (**c**) Punch 3 with a radius of 3.0 mm coated with PVD AlTiCrN.

**Figure 15:**
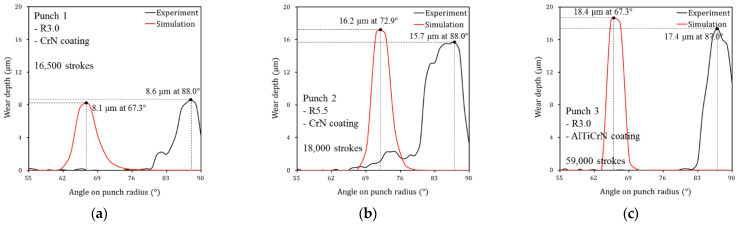
Wear depth on the punch radius obtained from experiments and simulations after the failure: (**a**) Punch 1 with a radius of 3.0 mm coated with PVD CrN, (**b**) Punch 2 with a radius of 5.5 mm coated with PVD CrN, and (**c**) Punch 3 with a radius of 3.0 mm coated with PVD AlTiCrN.

**Figure 16:**
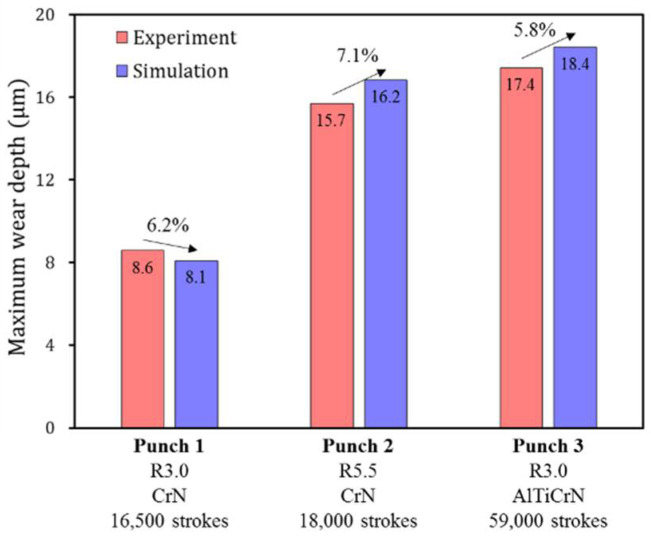
Difference in maximum wear depths between experiments and simulations.

Figure 13 confirms that the constructed wear simulation method can reliably predict the evolutionary wear behavior of sheet metal forming. The analytical solution and simulation results based on the revised Archard’s wear model were compared with the experimental results. It can be confirmed that the analytical solution and simulation results based on the revised Archard’s wear model accurately simulate the evolutionary wear behaviors of experimental results for each punch. The results obtained using the proposed wear simulation process are quantitatively reliable in predicting the rapidly increasing wear depth behavior after failure and in determining the number of strokes at which failure occurs. Figure 14 illustrates the predicted wear distributions and worn surface conditions on the punch radius for each punch condition after the failure; the predicted wear distribution confirms the amount and location of the computed wear depth. For all punches, predicted wear is concentrated on the punch radius. The wear of Punch 2 (Figure 14b) is more widely distributed than Punch 1 and Punch 3 (Figure 14a,c) because the shape of Punch 2 has a larger radius of 5.5 mm. The predicted wear is localized on the specific angular location, but punch surface conditions after the wear test show a long wear track in the sliding contact direction. The reason for this is that the wear is continuously propagated in the sliding contact direction after wear is initiated at the specific angular location. To check the accuracy of the amount and angular location of the predicted wear distribution, Figure 15 compares the wear depth for experimental and simulation results after the failure occurred. The wear location confirmed in the experiments is approximately 88° on the punch radius for all three punch conditions. However, the angular location of the maximum wear depth predicted from the simulation was approximately 15°–20° ahead of that of the experiments. As the punch stroke progresses, the steel sheet comes into contact with the punch at 55° first and then in the 90° direction. Therefore, it is inferred that during the stamping process, wear debris generated by the high contact pressure at about 70° of the punch radius moves in the 90° direction, and severe wear occurred at approximately 88° on the punch radius due to the wear debris. To verify the simulation accuracy, Figure 16 compares the difference in maximum wear depths between experiments and simulations. For each punch, the predicted maximum wear depth after the failure showed differences of 6.2%, 7.1% and 5.8%, respectively, from the experiments. Therefore, it was confirmed that the proposed wear simulation method is reliable in evaluating the wear depth and lifetime of stamping tools.

## 7. Discussion

A simplified Archard’s wear model was compared with experimental results of CrN-coated punches to confirm the limitations of predicting evolutionary wear behavior in sheet metal forming. The elemental form of the simplified Archard’s wear model for CrN-coated punches was fitted using experimental wear data, subsequently; the dimensionless wear coefficient of 0.074 was obtained, as follows:

(8) $$w_{CrN}=\frac{0.074}{2105}\int \left(p_{t}v_{t}\right)dt.$$

**Figure 17:**
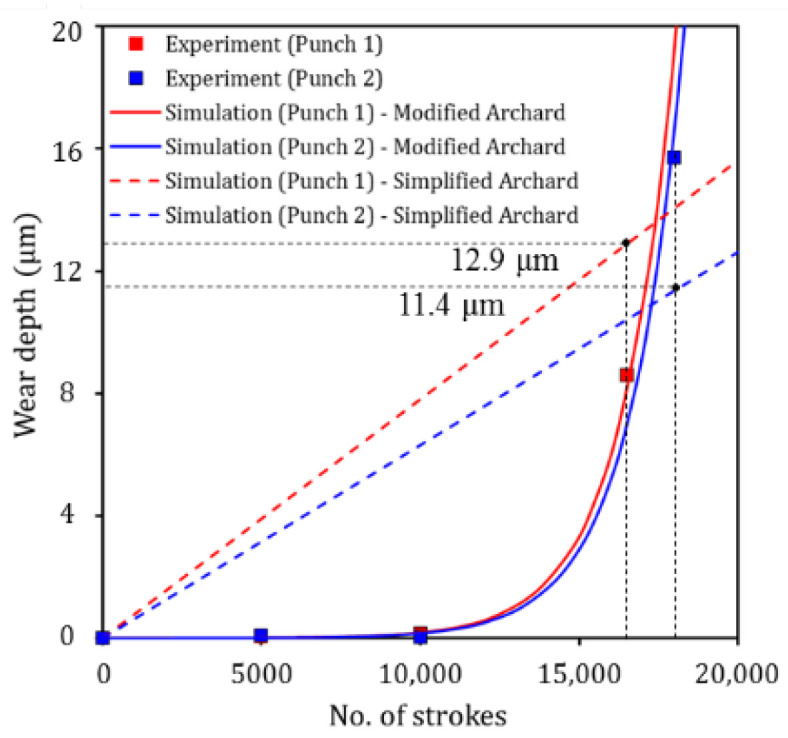
Simulation results based on simplified Archard’s wear model for CrN-coated punches.

The simulation results based on Equation (8) were obtained for CrN-coated punches and compared with experimental and simulation results based on the modified Archard’s wear model, as shown in Figure 17. Punch 1 (CrN-coated punch with a radius of 3.0 mm) failed after 16,500 strokes and the wear depth measured was 8.6 μm. However, the predicted wear depth based on the simplified Archard’s wear model is 12.9 μm. For Punch 2 (CrN-coated punch with a radius of 5.5 mm), failure occurred after 18,000 strokes, and the wear depth measured was 17.4 μm. The predicted wear depth at 18,000 strokes based on the simplified Archard’s wear model was 11.4 μm. It can be observed that wear depth predicted by the simplified Archard’s wear model in terms of the number of strokes at which failure occurred, significantly differed from experimental results. In addition, the experimental and simulation results based on the modified Archard’s wear model demonstrated no wear at 5000 and 10,000 strokes (i.e., before failure). It was confirmed that the simplified Archard’s wear model has limitations in simulating nonlinear wear behavior of the tool in sheet metal forming.

**Figure 18:**
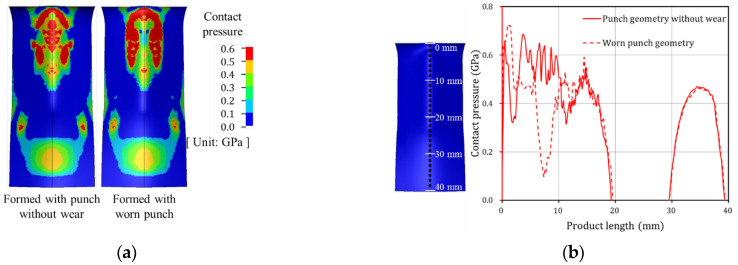
Contact pressure of the product formed with punch geometry without wear and worn punch geometry: (**a**) contour plot of contact pressure, (**b**) quantitative comparison of contact pressure.

**Figure 19:**
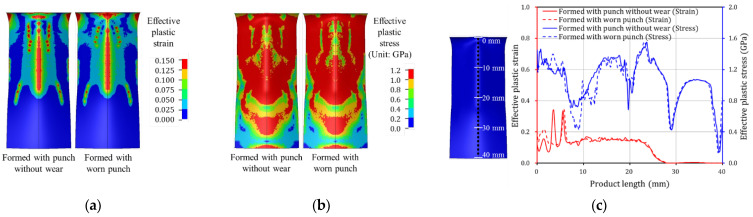
Effective plastic strain and stress of the product formed with punch geometry without wear and worn punch geometry: (**a**) contour plot of effective plastic strain, (**b**) contour plot of effective plastic stress, (**c**) quantitative comparison of effective plastic stress and strain.

**Figure 20:**
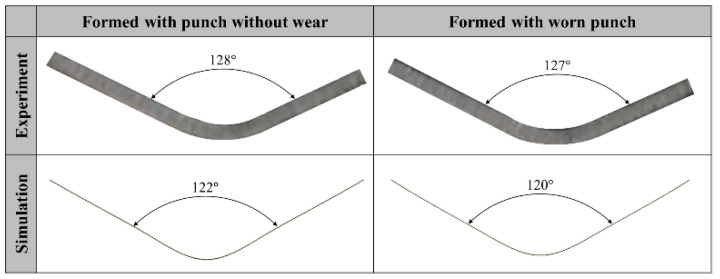
Comparison of experiment and simulation results for the cross-section of the product formed with punch geometry without wear and worn punch geometry.

Tool wear during the press forming process leads to reduced product quality. From the results of the Punch 1 wear test, it was confirmed that although the wear depth was 8.6 μm, severe scratches occurred on the product surface. The contact pressures of the formed product were compared when formed with punch geometry without wear and with worn punch geometry. Figure 18a shows the contact pressure of the formed product before and after failure initiation. It can be observed that the contact pressure before failure is higher than that after failure in the middle region of the product in contact with the curvature region of the punch. Figure 18b plots the contact pressure in the middle region of the product (dotted line), which is in contact with the punch curvature region. The contact pressure of the product formed prior to the failure (red solid line) is higher than that post failure (red dash line) in a product length of approximately 3 to 10 mm. To confirm the effect of this minor wear depth on product deformation shapes, the effective plastic strain and stress of the formed products before and after failure was analyzed. The effective plastic strain and stress of the formed products were compared when formed with punch geometry without wear and with worn punch geometry. Figure 19a shows the effective plastic strain of the formed product before and after failure. It can be observed that the effective plastic strain prior to failure is higher than that after failure in the middle region of the product in contact with the curvature region of the punch. As shown in Figure 19b, the effective plastic stress before failure is also higher in the middle region of the product. To quantitatively compare product conditions before and after failure, Figure 19c plots the effective plastic strain and stress in the middle region of the product (dotted line), which is in contact with the punch curvature region. The effective plastic strain of the product formed with punch geometry without wear (red solid line) is approximately 0.35 in the range of approximately 3 to 4 mm in product length, which is twice as high as that after failure (red dash line). The effective plastic stress of the product formed prior to failure (blue solid line) is higher than that post failure (blue dash line) in a product length of approximately 7 to 13 mm. The effective plastic strain and stress of the product decreased after failure because the radius of the curvature region of the punch increased owing to wear. Figure 20 compares experimental and simulation results for the cross-section of the product formed before and after failure. For the experimental results, the cross-sectional angle of the product formed before failure was 128°, however, this decreased to 127° once failure occurred. Simulation results also demonstrated that the cross-sectional angle of the product formed with punch geometry without wear was 122°, however, the cross-sectional angle of the product formed with worn punch geometry was reduced to 120°. It is inferred that because the effective plastic stress of the formed product decreases after failure, the elastic recovery and cross-sectional angle of the product decreases. Because the products used in this study are small, the geometric errors owing to springback are insignificant. However, larger parts such as automobile components may exhibit significant geometric errors owing to tool wear. Therefore, tool wear must be more effectively managed to improve product surface quality and geometrical accuracy.

## 8. Conclusions

An efficient wear simulation method was developed to predict quantitative wear reasonably in reduced computational time. The following conclusions are drawn from the results of this study.

In the stamping of a 1.2-mm-thick uncoated TRIP1180 steel sheet, there are no noticeable changes in the punch surface profile before failure initiation. However, after failure, the wear depth rapidly increases. Punch 1 (R3.0, CrN), which failed at 16,500 strokes, had less wear resistance than Punch 2 (R5.5, CrN), which failed at 18,000 strokes, because the former has a higher contact pressure than the latter. Punch 3 (R3.0, AlTiCrN) exhibited wear response at 59,000 strokes and, thus, demonstrated significantly improved wear resistance over Punch 1 (R3.0, CrN) because the AlTiCrN coating has a higher hardness than the CrN coating. By considering the punch wear characteristics, a nonlinear equation from a modified form of Archard’s wear model was proposed to predict the nonlinear wear behavior considering punch shapes (contact pressure) and coating hardness. The proposed wear model is suitable for predicting the rapidly increasing wear depth behavior after failure and determining the number of strokes required to achieve the desired wear depth. The geometry update and accumulated wear from the previous iteration of the wear simulation are rendered unnecessary by utilizing the scale factor when implementing the wear simulation method. Thus, the proposed method can decrease the computational time required for the simulation.

## Figures and Tables

**Table 1:** Summary of the mechanical properties of a TRIP 1180 steel sheet obtained using uniaxial tension tests [35].

YS ^1^ (MPa)	UTS ^2^ (MPa)	R-Value			Swift Hardening Law $\sigma =k{\left(\varepsilon_{0}+\varepsilon \right)}^{n}$		
		0°	45°	90°	*k* (MPa)	$\varepsilon_{0}$	n
932.8	1195.9	0.7382	0.9786	0.8671	1584.6	0.0036	0.0858

^1^ YS: yield stress; ^2^ UTS: ultimate tensile stress.

**Table 2:** Summary of the mechanical properties of D2 tool steel [39].

Modulus of Elasticity	203 GPa
Yield stress	411 MPa
Ultimate tensile stress	758 MPa
Modulus of toughness	81 MPa
Fracture stress	723 MPa
Fracture strain	1.97%

**Table 3:** Chemical compositions of TRIP1180 steel and STD-11 tool steel (wt%).

	C	Si	Mn	Cr	Mo	V	P	S
TRIP1180	0.285	1.61	2.15	-	-	-	0.018	0.001
STD-11	1.55	0.26	0.30	11.36	0.81	0.20	-	-

**Table 4:** Summary of stamping punch conditions for the wear test.

Punch Number	Punch Material	Punch Shape	Coating
1		R3.0	PVD CrN
2	STD-11	R5.5	PVD CrN
3		R3.0	PVD AlTiCrN

**Table 5:** FE model details and computational time for each element size case.

	Punch	Blank	Pad	Die	Computational Time
Element size (mm)	0.2	0.156 (lv. 6)	0.5	0.5	8 h
	0.4	0.313 (lv. 5)			2 h
	0.8	0.625 (lv. 4)			20 min
Element type	Rigid body	Deformable shell	Rigid body		

**Table 6:** The anisotropic parameters of the Hill’s 1948 yield criteria used in the simulation.

Anisotropic Parameters			
F	G	H	N
$5.629\;\times \;10^{-7}$	$6.612\;\times \;10^{-7}$	$4.881\;\times \;10^{-7}$	$1.810\;\times \;10^{-6}$

## Data Availability

The data presented in this study are available on request from the corresponding author.
